# The Therapeutic Value of Animal Extracts*Read before the Brazos Valley Medical Association, at Cameron, Texas, May 12 and 13, 1903.

**Published:** 1903-09

**Authors:** A. S. Epperson

**Affiliations:** Cameron, Texas


For Texas Medical Journal.

### The Therapeutic Value of Animal Extracts.*

BY A. S. EPPERSON, M. D., CAMERON, TEXAS.

Under this heading are included not only the extracts of various tissues at present utilized in therapeutics, but likewise the tissues themselves, and all the preparations, active principles, etc., obtained from them. Of the animal tissues and their products employed therapeutically, the ductless glands whose functions are now known to be intimately associated with metabolism have by far taken the lead over all other portions of the animal organism utilized. And if they continue to increasingly engage attention, as they have of late, the time is not far distant when antitoxines will find in them a potent rival. In this attempt to interest this able body of medical men, I will not give a re-hash of my experience, though it has been limited. During the past three or four years I have not missed an opportunity to prescribe these remedies where I thought them indicated and have been well pleased with the results.

To my mind, some of these extracts form a most rational treatment for a number of the most common diseases of our every-day practice.

Extract of thyroid gland is my favorite of the animal extracts, because I found it valuable in many diseases, not only in myxœdema and cretinism, but in melancholia and the mental conditions connected with the menopause, some of the cutaneous diseases, backward growth and delayed dentition in children. Children with dirty complexions, morbid appetites, coarse, dry hair and dull of intellect are benefited by thyroid extract. Neurasthenia, in both children and adults, improves under this treatment in connection with nerve tonics.

In old people, with weak hearts, it is well to give thyroid extract with great care, keeping the patient under direct supervision. I usually begin with one grain of the powder (Parke, Davis & Co.'s

---

*Read before the Brazos Valley Medical Association, at Cameron, Texas, May 12 and 13, 1903.

preparation), three times a day and gradually increase it to tolerance.

It doesn't seem to be a great heart-depressant, but produces the sensation of tightness and oppression in the chest and palpitation of the heart.

I have under treatment at present a case of myxœdema in a lady 51 years of age. She had been an invalid ten years. When she began taking thyroid eight months ago, her heart was very weak and at first she could take only one-half grain three time a day and has never been able to take more than three grains a day, but she improved rapidly and is at present doing her own cooking; and strange to say, she is cutting her wisdom teeth, which should have erupted twenty or more years ago. In this case the myxœdema came on after an attack of scarlet fever, which caused atrophy of the thyroid gland. She lost her hearing at the same time, which she has never recovered. I have used extract of thyroid gland in obesity, but am of the opinion that we have remedies superior to it in reducing body weight and adipose tissue.

My experience with suprarenal extract has also been quite pleasing. I have found it to be an exceedingly energetic vaso-constrictor and has proven valuable in hemorrhages of internal organs, the lungs and stomach especially, intestine, kidneys, uterus also, as well as in external mucous membrane and to prevent hemorrhage in operations of mucous membrane. I have never seen the administration of this extract accompanied with harmful or unpleasant effects. My experience with other of the animal extracts has been too limited to be interesting, though preparations of the various organs, as the lungs, spleen, ovaries and mammary glands, bone-marrow, brain and nerves, liver, pancreas and testicles have all been used with sufficient success to merit the confidence of the medical profession.

